# Evaluation of the adverse events following immunization surveillance system in Guruve district, Mashonaland Central 2017

**DOI:** 10.11604/pamj.2018.31.202.16573

**Published:** 2018-11-22

**Authors:** Mutata Constantine, Tshuma Cremance, Tsitsi Patience Juru, Shambira Gerald, Gombe Tafara Notion, Nsubuga Peter, Tshimanga Mufuta

**Affiliations:** 1 University of Zimbabwe, Department of Community Medicine, Zimbabwe; 2 Provincial Medical Director, Mashonaland Central Province, Zimbabwe; 3 Global Public Health Solutions, Atlanta, GA, USA

**Keywords:** AEFI, surveillance, Guruve, Zimbabwe, vaccination

## Abstract

**Introduction:**

An adverse event following immunisation is any untoward medical occurrence which follows vaccination. Frequency of adverse events ranges from 13% to 34% and they should be reported regardless of severity. From the beginning of 2016 to mid-2017, Guruve district in Zimbabwe did not report any AEFIs. This suggests the surveillance system may be failing to detect adverse events. We therefore evaluated the AEFI surveillance system in Guruve district.

**Methods:**

We conducted a surveillance system evaluation using the updated Centers for Disease Control guidelines for evaluating public health surveillance systems. We interviewed health workers and caregivers of babies under 2 years in Guruve district. We also reviewed all records on AEFI surveillance for the period of January 2016 to November 2017.

**Results:**

We recruited 31 health workers and 33 caregivers into the study. Between January 2016 and mid-2017, 39% of the caregivers had children who had suffered AEFIs and 45% of the health workers had encountered AEFIs but none had been notified. The main reasons for failure to report AEFIs included health workers' fear of personal consequences and caregivers thinking that an adverse event was not serious enough to report. Knowledge of the surveillance system was good amongst the majority of health workers. All the resources needed by the surveillance system were available.

**Conclusion:**

We concluded that health workers in Guruve district were afraid to report adverse events following immunization and caregivers were reluctant to report mild adverse events hence the surveillance system was performing poorly and was not useful. However, the stability of the system and the good knowledge gives a good foundation for improving the surveillance system.

## Introduction

An adverse event following immunisation (AEFI) is any untoward medical occurrence which follows vaccination and does not necessarily have a causal relationship with the usage of the vaccine [1]. Adverse events range from mild to severe, and the mild events include fever, pain at injection site and local swelling. Severe reactions can include convulsions, coma and even death [2]. The majority of adverse events following immunisation are mild and resolve quickly, but one cannot predict individuals who might have a mild or serious reaction to a vaccine [3]. AEFIs may be true adverse reactions that are intrinsic to the vaccine, caused by the way it is administered, related to an underlying condition in the recipient, or coincidental. The World Health Organization (WHO) classifies AEFIs into four main categories which are programme related, vaccine-induced, coincidental and unknown [1]. A clinical trial conducted by Decker et al. in the USA showed that mild adverse events such as fever occurred in more than 35% of children who were vaccinated [4]. In 2011, WHO came up with the global vaccine safety initiative (GVSI) whose primary objective is AEFI detection. The GVSI aims to ensure that monitoring of vaccine safety takes place even in low resource settings [1]. The Global Advisory Committee on Vaccine Safety (GACVS) proposed a reporting rate of at least 10 severe AEFIs per 100,000 surviving infants and this is the vaccine safety indicator which was adopted by WHO [5]. There is no indicator for minor AEFIs.

In the WHO African region, only half of the countries (i.e. 51%) reported AEFIs in 2015 and Africa had the second lowest case reporting rate of 74 per 100,000 surviving infants while the Eastern Mediterranean Region had the highest rate of 2,740 per 100,000 surviving infants [6]. A study conducted in Mali and Gambia showed that in children who received the meningococcal A vaccine 13.4%-34.3% developed minor adverse events [7]. According to the WHO, all adverse events that are of concern to the caregiver should be reported regardless of severity [8]. Allegations that vaccination causes adverse events must be dealt with rapidly and effectively. Failure to do so can undermine confidence in a vaccine and ultimately have dramatic consequences for immunisation coverage [1]. Zimbabwe introduced an AEFI surveillance system in 2000 which is based on the WHO guidelines [9]. The system is meant to identify and correct programme errors and distinguish coincidental events from true AEFIs. Mashonaland Central was expected to report at least six severe AEFIs from the beginning of 2016 to mid-2017 based on its infant survival rate; they only managed two. From the beginning of 2016 to mid-2017, Guruve district in Mashonaland Central did not report any AEFIs. This lack of reports suggests that the AEFI surveillance system may be failing to detect adverse events. Failure to detect adverse events that occur in the community may result in loss of confidence in the safety of vaccines [10, 11]. There is no published evidence of previous evaluation of this surveillance system; it is for this reason that we evaluated the AEFI surveillance system in Guruve district to identify reasons for its poor performance and make recommendations to improve it.

## Methods

**Figure 1:**
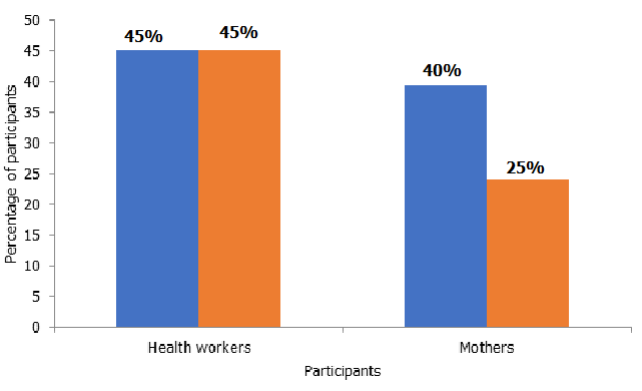
The AEFI surveillance system flow diagram, Zimbabwe

**Flow of information:** Guruve district uses a passive surveillance system where caregivers detect adverse events in their children in the community. The caregivers then report to the clinic where the nurse confirms the AEFI, manages and reports the AEFI to the district level within 24 hours. When an AEFI is reported, five notification forms are completed at the clinic, four are sent to the district, and one is filed at the clinic. When the district is notified they facilitate case management and investigation of the AEFI. They also notify the provincial level within 24 hours and send three of the notification forms to them. The fourth form is filed at the district level. The province supports investigations of serious AEFIs, and they notify the national level and Medicines Control Authority of Zimbabwe (MCAZ). The national level reviews AEFI case reports and gives regular feedback to lower levels. The MCAZ reviews all AEFIs at the national committee meeting and provides feedback to the reporter through the same channels (Figure 1).

**Study design:** We conducted a surveillance system evaluation using the updated Centers for Disease Control (CDC) guidelines for evaluating public health surveillance systems in November 2017.

**Study setting:** The study was conducted at health facilities in Guruve district, Mashonaland Central. Guruve district has a population of 124,000 (census 2012), 96% of which is rural. The population is predominantly young with children < 9 years old constituting 29% of the population. The main economic activity is agriculture, small-scale and commercial. There are 21 health facilities in Guruve, a district hospital and 20 clinics. All the facilities offer vaccination services at the sites but some communities are hard to reach due to distance or geographical barriers and sometimes outreach programmes are conducted to cater to them.

**Study population:** We interviewed healthcare workers, key informants and parents of children < 2 years old who had been vaccinated at least once in Guruve district. We also reviewed all records on AEFI surveillance for the period of January 2016 to November 2017.

**Sample size:** Based on a study by Muchekeza *et al*. in Kwekwe district where 98% of the health workers perceived the AEFI surveillance system to be useful, the Dobson formula

$$n=Z^{2}\cdot p\left(1-p\right)/d^{2}$$

Where Z = 1.96, p = 0.98 and d = 0.05 was used to calculate a minimum sample size of 31. In the same study, 2% of the caregivers had no formal education, using the same formula, the calculated sample size for caregivers was 31.

**Sampling:** Guruve District has 21 health facilities, one district hospital and 20 clinics. We purposively selected the district hospital into the study since it is the largest health facility in the district serving the largest population and 10 out of the 20 clinics were randomly selected into the study using a random number generator (Random lite^TM^ version 1.2). The clinics were assigned numbers 1 to 20 in alphabetical order, and a random number was generated with replacement until the sample size was satisfied. At each clinic nurses who were found to be on duty on the day of data collection were recruited into the study, at the hospital six nurses (i.e., three from the outpatient's department and children's ward respectively) were randomly selected from those found on duty on the day of data collection. Caregivers were recruited from the same facilities, a maximum of three from each facility were randomly selected amongst those who had visited the facility for immunisation services on the day of data collection. All available records were reviewed.

**Data collection:** Separate interviewer-administered questionnaires were used to collect information from the healthcare workers and caregivers. The health workers were interviewed to assess their knowledge of the AEFI surveillance system as well to establish its perceived simplicity, acceptability, stability and usefulness. The caregivers' knowledge of the surveillance system was also assessed as well as their reasons for not reporting AEFIs. Key informant interviews were conducted with the provincial EPI manager, the District Medical Officer (DMO), District Nursing Officer (DNO) and community nurse in charge of immunisation for Guruve. A checklist was used to objectively assess all available AEFI reporting forms regarding completeness, simplicity, data quality and timeliness.

**Data management analysis:** We created a questionnaire in Epi Info 7^TM^ and captured all the collected data. We used the software for data analysis to generate means, proportions and frequencies.

### Measurement of variables

**Usefulness:** Usefulness of an AEFI surveillance system measures the system's ability to contribute to the detection and prevention of adverse events as well as contributing to new knowledge about the occurrence of AEFIs. Respondents were asked what the collected data is used for, and what public health actions or decisions have been carried out or made based on the findings from data collected by the surveillance system. Records of meetings were reviewed to see if AEFIs were discussed in meetings and if decisions were made based on AEFI data. The summary of all other attributes was also used to assess usefulness.

**Simplicity:** Simplicity refers to the structure and the ease of operation of the surveillance system. It was assessed by looking at the proportions of health workers who could accurately describe the operation of the system, know the case definition and describe the completion of case investigation forms as easy and not time-consuming. The need for special training was used in assessing simplicity and capacity to complete forms in a specified period.

**Acceptability:** Acceptability refers to the willingness of persons and organisations to participate in the surveillance system. It was determined by looking at the completeness of reporting forms and timeliness of reporting. We also compared the number of adverse events that each health worker and caregiver identified during the period under study and the number of times that they reported.

**Sensitivity:** Sensitivity refers to the proportion of AEFIs that occurred that were picked up by the surveillance system; it can also refer to the ability of the system to monitor changes in the number of cases over time. It was measured by comparing the number of cases that were detected to those reported to have occurred in the community by the caregivers. It was also compared to the recommended WHO case detection rate of 10 per 100,000 surviving infants.

**Timeliness:** Refers to the speed at which data is transmitted between different levels in the surveillance system. To answer the research question, the time taken for caregivers to report an adverse event or a health facility and the time taken for health workers to report to the district were evaluated.

**Stability:** Stability refers to the reliability (i.e. the ability to collect, manage, and provide data adequately without failure) and availability (the ability to be operational when it is needed) of the surveillance system. This was assessed by looking at the availability of reporting forms and the availability of a health worker trained to identify and report AEFIs at each facility every time.

**Knowledge:** Health care workers' knowledge of the AEFI surveillance system was assessed by asking them the meaning of AEFI, to name five examples of common adverse events, to describe the reporting channels and the timelines for reporting. Caregiver knowledge was also assessed since they are the ones who are supposed to report any AEFIs to the health facility. They were asked to name any three things that could be of concern to them after immunisation, what they would do if they were worried about anything and how soon they should take this action. A Likert type scale was used to rate knowledge. Getting 0 to 2 questions correct was scored as poor knowledge, 3-4 was fair, and 5 was good knowledge.

**Permission and ethical considerations:** Permission to carry out the study was sought from the Provincial Medical Director for Mashonaland Central province, the district medical officer for Guruve district and Health Studies Office. We obtained informed written consent from all study participants. Study participants were not coerced or offered rewards for participating in the study. All interviews were held in privacy. Names of participants were not recorded and any information gathered will remain confidential.

## Results

**Demographic characteristics of study participants:** We successfully recruited 31 health workers and 33 caregivers into the study. The majority of health workers who participated in the study were female 22/31 (71%) and primary care nurses 21/31 (68%) with median years in service of 6 years (Table 1). All the caregivers that were recruited into the study were mothers. Their median age was 28 (Q_1_= 22; Q_3_ =32) and their median number of children was 2 (Q_1_ = 2; Q_3_ = 4).

**Table 1:** Demographic characteristics of health workers and reasons for not reporting AEFIs in Guruve District, Mashonaland Central 2017

Characteristics	Frequency (%) (n=31)
Gender	
Female	22 (71)
Qualification	
Midwife	4 (13)
Registered general nurse	6 (19)
Primary care nurse	21 (68)
Median years in service	6 (Q_1_=1 Q_3_ =10)
**Health worker reasons for not reporting**	**Frequency (%) n=31**
Afraid of personal consequences	14 (45%)
Caregivers not reporting	10 (32)
Do not know how to report	5 (16)
Overwhelmed by work	1 (3)
AEFIs are not occurring	1 (3)

**Reasons for not notifying AEFIs:** All the health workers were asked what they thought were the reasons why AEFIs were not being notified. The majority of health workers 14/31 (45%) said that adverse events were not being reported because they were afraid of the personal consequences of reporting. These included fear of being blamed for causing adverse events and being investigated. Thirty-two per cent (10/31) of the health workers said that while adverse events may be occurring in the communities, they were not being notified because caregivers were not reporting them to health facilities (Table 1).

**Table 2:** Simplicity of the AEFI surveillance system in Guruve District 2017

Variable	Response	Frequency (%)
Encountered an AEFI	Yes	14/31 (45)
Ever completed an AEFI notification form	Yes	10/31 (32)
Did you find the forms difficult to complete	Yes	2/10 (20)
Encountered an AEFI but did not report because they did not know how to report	Yes	3/14 (21)
Need training to identify AEFIs	Yes	15/31 (48)
Need training to complete AEFI notification forms	Yes	26/31 (84)

**Sensitivity:** Compared to the recommended WHO case detection rate of 10 serious cases per 100 000 surviving infants, Guruve district identified zero cases from January 2016 to November 2017. Of the interviewed caregivers, 13/33 (39%) had children who had experienced AEFIs while 14/31 (45%) of the health workers had encountered AEFIs during the period under study. The surveillance system did not pick all these cases. The actual burden of AEFIs could not be determined.

**Figure 2:**
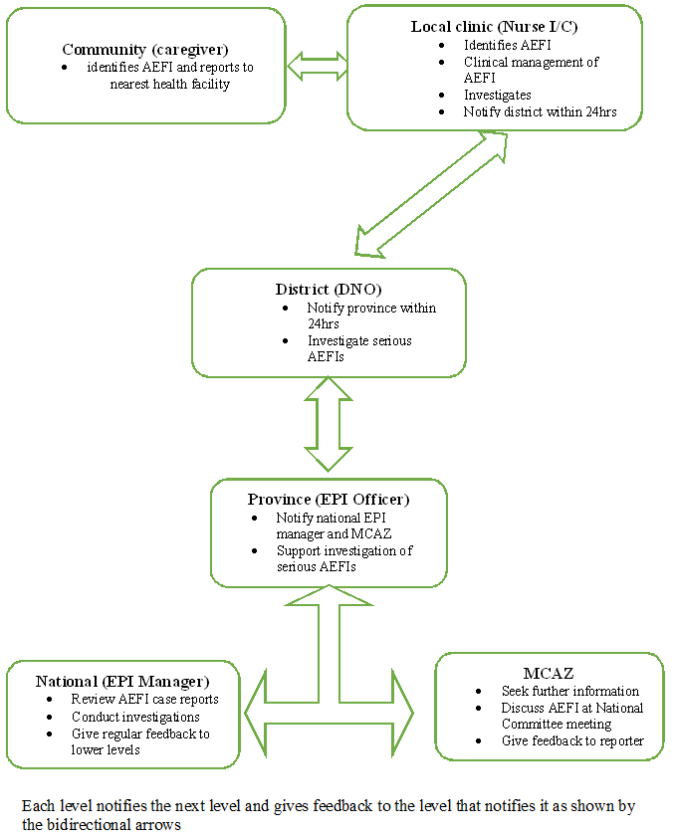
Participants who encountered AEFIs in 2017 in Guruve District

**Acceptability:** All of the health workers reported that it was their duty to notify AEFIs and they were willing to participate in the surveillance system. Although 14/31 (45%) of health workers said they had encountered AEFIs during the period under study, there was no evidence of completed forms at all the health facilities, and there were no line lists of the AEFIs they had encountered (figure 2). All the caregivers said they were willing to report severe adverse events to their health facilities. However, 31/33 (94%) of them said they would not report if they did not perceive the adverse event to be severe. Of the 33 caregivers who were interviewed, 13 (39%) of them had children who had experienced AEFIs, and 8 (65%) of them did not report them to health facilities (Figure 2).

**Simplicity:** Fourteen (45%) of the interviewed health workers had ever encountered an AEFI and three (21%) of them found the AEFI notification form challenging to complete. Twenty-six (85%) healthcare workers said they needed the training to complete the notification forms (Table 2). Three caregivers who had experienced adverse events with their children were asked to report the events to health workers to assess the time it took to complete the notification forms. All three health workers took between 10 and 20 minutes without any difficulties.

**Knowledge of the AEFI surveillance system:** All the healthcare workers knew where to send the completed notification forms (Table 3). The least known variable was the number of notifying forms that must be completed (55%). Two-thirds (22) of the interviewed caregivers had ever heard of adverse events following immunisation, and all of the caregivers knew that they should report to a health facility if anything untoward happened after vaccination. Knowledge was found to be good amongst 15 (48%) of the health workers and 15 (45%) of the caregivers. Twelve (36%) of the caregivers had poor knowledge.

**Table 3:** Knowledge of the AEFI surveillance system in Guruve District, 2017

Health care workers’ knowledge	Frequency (%) n=31
	Yes
Knows meaning of AEFI	28 (90)
Knows at least 3 examples of AEFIs	29 (94)
Knows number of notifying forms to be completed	17 (55)
Knows where to send completed forms	31 (100)
Knows how long it should take to notify the next level	25 (81)
**Caregivers’ knowledge**	**Frequency (%) n=33**
	**Yes**
Had ever heard of AEFIs	22 (66)
Can name 2 examples of AEFIs	20 (61)
Nurses explained AEFIs during Immunization	20 (61)
Knew to return to clinic if an AEFI occurred to their child	33 (100)

**Stability:** All sampled health facilities had a working EPI fridge for vaccine storage and had guidelines on how to identify, manage and notify adverse events. All the health facilities had at least one health worker trained in AEFI surveillance, and they all had notification forms on site. A working telephone was available at each health facility to notify the district in the event of an AEFI being identified. Of the interviewed caregivers, 20 (61%) of them had been taught by the health workers on the need to report to the health facility if an adverse event occurred.

**Timeliness:** Since there was no notification being done in all the health facilities, the timeliness of the surveillance system could not be objectively assessed. The majority of health workers 26/31 (84%) knew the correct time recommended for notifying the next level (i.e., 24 hours).

**Usefulness of the system:** The majority of the health workers 29/31 (94%) perceived the AEFI surveillance system in Guruve district to be useful. However, only 8 (26%) said they had ever discussed AEFIs in regular meetings, and 3 (10%) said they had implemented new practices as a result of AEFI surveillance data. On records review, it was found that no health facility had any record of having discussed AEFIs in meetings or using AEFI surveillance data to influence policy or clinical practice.

## Discussion

We found that the health workers were not reporting adverse events following immunisation despite having good knowledge of the surveillance system. Most of them were afraid to report AEFIs because they feared they would be investigated and face personal consequences if the adverse event was found to be a result of an error on their part. This fear of reporting was also reported in studies in South Africa and Kenya [12, 13]. This persistent fear could be a result of insufficient training and lack of supportive supervision [14]. It can also be understood in an environment where health workers feel their job security is not guaranteed. We also found that caregivers of children were not reporting AEFIs to health facilities especially when they perceived them to be mild. This was despite most of them knowing that they should report adverse events to the clinic. Our finding is contrary to what Muchekeza *et al.* published in Kwekwe district of Zimbabwe where caregivers had a low reporting rate because they had inadequate knowledge of the surveillance system [15]. This difference shows that caregiver knowledge is not sufficient to improve their reporting rate and there is need to address the inconvenience of travelling back to the clinic. There is need to consider complementing the passive surveillance system with a participant-centred active surveillance system where caregivers can send an SMS or use other communication technologies such as WhatsApp. Such innovations have been tried in low resource countries such as Cameroon and Cambodia with notable improvement in AEFI reporting [16, 17]. The fear of reporting for health workers, coupled with the inconvenience of travelling back to the clinic for caregivers, made the surveillance system not acceptable. All the health workers who encountered AEFIs during the period under study did not report them, and the majority of caregivers said they would not report an adverse event if they thought it was mild. Mild adverse events have the potential to become severe if they are not reported, which may result in the community losing confidence in vaccines [1].

The sensitivity of the surveillance system was poor due to attitudes of caregivers and health workers towards reporting which suggests inadequacies in community sensitisation and training of health workers. We found knowledge among the health workers and caregivers to be good. At every health facility in Guruve district, there was at least one nurse formally trained in AEFI surveillance, and this gives the other nurses an opportunity to learn from the trained cadre, this is supported by results from a study in Kenya where peer mentorship resulted in improved knowledge [18]. At the clinics, we visited we found that every morning the caregivers who bring their children for vaccination are taught about adverse events by the nurse at the clinic. However, this knowledge did not translate into improved reporting of AEFIs. We also found the surveillance system to be simple as the case definition of an AEFI removes the need to ascertain causality, anything that a caregiver comes complaining about after vaccination is considered an AEFI. The skills needed to use the system also existed at each health facility. Our study was not without limitations. Respondents were asked to recall events that had happened in the past, and this may have introduced recall bias into the study. In anticipation of this bias, we only asked all our respondents to recall events of a period not exceeding 18 months. A healthcare worker administered the questionnaires, and this may have introduced social desirability bias on the part of caregivers. We conducted our study in a rural district, and the results of our study may not be generalizable to urban districts.

## Conclusion

We concluded that health workers in Guruve district were afraid to report adverse events following immunisation and caregivers were reluctant to report mild adverse events hence the surveillance system was performing poorly and was not useful. However, the stability of the system and the knowledge of both health workers and caregivers gives a good foundation for improving the surveillance system. We, therefore, recommended retraining health workers focusing on the importance of notifying all adverse events and allaying their fears of reporting. We also supported the introduction of a participant-centred active surveillance system where caregivers can use mobile phones to notify adverse events to the health facility. This reporting can be done via Short message services (SMS), social media platforms or an unanswered call that will be followed up by the health workers at the clinic.

### What is known about this topic

- Poor performance of the AEFI surveillance system was found to be a result of insufficient health worker and caregiver knowledge in a study in Kwekwe District of Zimbabwe;
- Evaluation of a similar surveillance system in Zimbabwe found that shortage of stationary resulted in poor performance;
- In Harare district of Zimbabwe, it was found that lack of feedback reports from the regulatory authority was the main reason for not reporting AEFIs.

### What this study adds

- This study shows that a surveillance system can still perform poorly when resources and knowledge are adequate;
- The study highlights the need to have a complimentary active surveillance system which would identify cases in the community as it was found that caregivers are reluctant to report AEFIs;
- We also show that fear of reporting is the major reason for the poor performance of the surveillance system in a rural district, which is contrary to what has been reported in urban districts.

## Competing interests

The authors declare no competing interests.

## References

[1] WHO Global Vaccine Safety.

[2] Zvanaka S, Tsitsi J, Chonzi P, Shambira G, Gombe NT, Tshimanga M (2017). Evaluation of the adverse events following immunizations surveillance system in Harare City, Zimbabwe, 2016: a descriptive cross sectional study. Pan African Medical Journal.

[3] General Practice Notebook Adverse events following immunisation (AEFIs).

[4] Edwards KM, Meade BD, Decker MD, Reed GF, Rennels MB, Steinhoff MC (1995). Comparison of 13 Acellular Pertussis Vaccines: overview and serologic response. Pediatrics.

[5] WHO Immunization, Vaccines and Biologicals. SAGE issues its 2018 assessment report of the Global Vaccine Action Plan.

[6] Lei J, Balakrishnan MR, Gidudu JF, Zuber PLF (2018). Use of a new global indicator for vaccine safety surveillance and trends in adverse events following immunization reporting 2000-2015. Vaccine.

[7] Sow SO, Okoko BJ, Diallo A, Viviani S, Borrow R, Carlone G (2011). Immunogenicity and Safety of a Meningococcal A Conjugate Vaccine in Africans. New England Journal of Medicine.

[8] London School of Hygiene and Tropical Medicine (2009). The use of epidemiological tools in conflict-affected populations: open-access educational resources for policy-makers. Types of surveillance.

[9] Ministry of Health and Child Care (2017). Adverse Events Following Immunization Surveillance Guidelines.

[10] Kibombo R, Asiimwe D, Matsiko J (2006). Vaccine safety perceptions among parents in developing countries and influence of adverse events following immunization (AEFI) on their decisions to vaccinate children.

[11] Mehta U, Milstien JB, Duclos P, Folb PI (2000). Developing a national system for dealing with adverse events following immunization. Bull World Health Organ, Bull World Health Organ.

[12] Graham JE, Borda-Rodriguez A, Huzair F, Zinck E (2012). Capacity for a global vaccine safety system: the perspective of national regulatory authorities. Vaccine.

[13] Masika CW, Atieli H, Were T (2016). Knowledge, perceptions and practice of nurses on surveillance of adverse events following childhood immunization in Nairobi, Kenya. Biomed Res Int.

[14] Samsiah A, Othman N, Jamshed S, Hassali MA (2016). Perceptions and attitudes towards medication error reporting in primary care clinics: a qualitative study in Malaysia. PLoS One.

[15] Muchekeza M, Chimusoro A, Ncube N, Pomerai KW (2014). Adverse events following immunisation (AEFI) surveillance in Kwekwe District, Midlands Province, Zimbabwe, 2009-2010. Journal of Vaccines and Vaccination.

[16] Cashman P, Macartney K, Khandaker G, King C, Gold M, Durrheim DN (2017). Participant-centred active surveillance of adverse events following immunisation: a narrative review. Int Health.

[17] Tsafack M, Ateudjieu J (2015). Improving community based AEFI (Adverse Events Following Immunization) reporting rate through telephone "beep" in a Cameroon health district: a randomized field trial. Pan Afr Med J.

[18] Ndwiga C, Abuya T, Mutemwa R, Kimani JK, Colombini M, Mayhew S (2014). Exploring experiences in peer mentoring as a strategy for capacity building in sexual reproductive health and HIV service integration in Kenya. BMC Health Serv Res.

